# Desensitization using IVIG alone for living-donor kidney transplant: impact on donor-specific antibodies

**DOI:** 10.1590/2175-8239-JBN-2021-0200

**Published:** 2022-04-08

**Authors:** Luiz Roberto de Sousa Ulisses, Jenaine Oliveira Paixão, Fabiana Agena, Patrícia Soares de Souza, Flávio J Paula, Gislene Bezerra, Hélcio Rodrigues, Nicolas Panajotopolous, Elias David-Neto, Maria Cristina Ribeiro de Castro

**Affiliations:** 1 Universidade de São Paulo, Hospital das Clínicas, Serviço de Transplante Renal, São Paulo, SP, Brasil.; 2 Universidade de São Paulo, Instituto do Coração da São Paulo, Laboratório de Imunologia, São Paulo, SP, Brasil.

**Keywords:** Antibodies, HLA Antigens, Living Donors, Graft Rejection, Histocompatibility Testing, Kidney Transplantation, Anticorpos, Antígenos HLA, Doadores Vivos, Rejeição de Enxerto, Teste de Histocompatibilidade, Transplante Renal

## Abstract

**Introduction:**

Sensitization to human leukocyte antigen is a barrier to. Few data have been published on desensitization using polyvalent human intravenous immunoglobulin (IVIG) alone.

**Methods:**

We retrospectively reviewed the of 45 patients with a positive complement-dependent cytotoxicity crossmatch (CDCXM) or flow cytometry crossmatch (FCXM) against living donors from January 2003 to December 2014. Of these, 12 were excluded. Patients received monthly IVIG infusions (2 g/kg) only until they had a negative T-cell and B-cell FCXM.

**Results:**

During the 33 patients, 22 (66.7%) underwent living donor kidney transplantation, 7 (21.2%) received a deceased donor graft, and 4 (12.1%) did not undergo transplantation. The median class I and II panel reactive antibodies for these patients were 80.5% (range 61%-95%) and 83.0% (range 42%-94%), respectively. Patients (81.8%) had a positive T-cell and/or B-cell CDCXM and 4 (18.2%) had a positive T-cell and/or B-cell FCXM. Patients underwent transplantation after a median of 6 (range 3-16). The median donor-specific antibody mean fluorescence intensity sum was 5057 (range 2246-11,691) before and 1389 (range 934-2492) after desensitization (p = 0.0001). Mean patient follow-up time after transplantation was 60.5 (SD, 36.8) months. Nine patients (45.0%). Death-censored graft survival at 1, 3, and 5 years after transplant was 86.4, 86.4, and 79.2%, respectively and patient survival was 95.5, 95.5, and 83.7%, respectively.

**Conclusions:**

Desensitization using IVIG alone is an effective strategy, allowing successful transplantation in 87.9% of these highly sensitized patients.

## Resumo

**Introdução:**

Sensibilização HLA é uma barreira ao transplante em pacientes sensibilizados. Há poucos dados publicados sobre dessensibilização utilizando somente imunoglobulina intravenosa humana polivalente (IgIV).

**Métodos:**

Revisamos retrospectivamente prontuários de 45 pacientes com prova cruzada positiva por citotoxicidade dependente do complemento (CDCXM) ou citometria de fluxo (FCXM) contra doadores vivos, de Janeiro/2003-Dezembro/2014. Destes, excluímos 12. 33 pacientes receberam infusões mensais de IgIV (2 g/kg) apenas até apresentarem FCXM células T e B negativa.

**Resultados:**

Durante dessensibilização, 22 pacientes (66,7%) realizaram transplante renal com doador vivo, 7 (21,2%) receberam enxerto de doador falecido, 4 (12,1%) não realizaram transplante. A mediana do painel de reatividade de anticorpos classes I e II para estes pacientes foi 80,5% (intervalo 61%-95%) e 83,0% (intervalo 42%-94%), respectivamente. 18 pacientes (81,8%) apresentaram CDCXM célula T e/ou B positiva; 4 (18,2%) apresentaram FCXM célula T e/ou B positiva. Pacientes realizaram transplante após mediana de 6 (intervalo 3-16) infusões. A mediana da somatória da intensidade média de fluorescência do anticorpo específico contra o doador foi 5057 (intervalo 2246-11.691) antes e 1389 (intervalo 934-2492) após dessensibilização (p = 0,0001). O tempo médio de acompanhamento do paciente pós transplante foi 60,5 (DP, 36,8) meses. Nove pacientes (45,0%) não apresentaram rejeição e 6 (27,3%) apresentaram rejeição mediada por anticorpos. Sobrevida do enxerto censurada para óbito em 1, 3, 5 anos após transplante foi 86,4; 86,4; 79,2%, respectivamente, e sobrevida do paciente foi 95,5; 95,5; 83,7%, respectivamente.

**Conclusões:**

Dessensibilização utilizando apenas IgIV é uma estratégia eficaz, permitindo transplante bem-sucedido em 87,9% destes pacientes altamente sensibilizados.

## Introduction

There is a group of patients who wait longer on the kidney transplant waiting list: sensitized patients. These patients develop anti-human leukocyte antigen (HLA) antibodies over time by previous blood transfusions, pregnancies, and/or transplants^1^. Sensitized patients have lower access to transplantation and are more susceptible to complications resulting from long-term dialysis, such as cardiovascular and infectious morbidity and mortality, in addition to loss of vascular and peritoneal access for dialysis. The difficulty in finding matching donors for sensitized patients among available deceased donors makes living donor transplant an option for these patients^2^.

The treatment administered to sensitized patients to improve their access to transplant is known as desensitization. However, desensitization protocols vary from center to center^3^. Generally, when a living donor is available, most centers use high-dose polyvalent human intravenous immunoglobulin (IVIG) combined with plasmapheresis sessions and immunosuppressive drugs that target B lymphocytes (rituximab), plasma cells (bortezomib), or cytokines (tocilizumab)^3^.

Few studies have used IVIG alone to enable kidney transplant with living donors^4^. Our goal was to show that the use of IVIG alone is an effective strategy for desensitization.

## Patients and Methods

### Study Design

Eligible participants were all sensitized patients aged ≥ 18 years who had a potential living donor with a positive complement-dependent cytotoxicity crossmatch (CDCXM) or flow cytometry crossmatch (FCXM). We retrospectively reviewed the medical records of 45 sensitized patients treated at the Kidney Transplant Service of Hospital das Clínicas (University of São Paulo) from January 2003 to December 2014. We collected the data up to December 30, 2017.

Of 45 patients, 12 were excluded. One patient died before starting the desensitization protocol and 11 were excluded during treatment: 4 patients decided to withdraw from the study, 4 switched to another transplant center, 1 was excluded due to donor withdrawal, 1 was subjected to a protocol that included apheresis and rituximab, and 1 found an identical donor after initiation of treatment.

Thirty-three patients remained in the study. Of these, 22 (66.7%) underwent a living-donor transplant, 7 (21.2%) underwent a deceased-donor transplant during treatment, 3 (9.1%) did not undergo a transplant until the end of the mean follow-up period of 60.5 (SD, 36.8) months, and 1 (3.0%) died during treatment.

The desensitization protocol consisted of IVIG therapy at a dose of 2 g/kg per month. For testing, the samples were collected after 3 weeks of IVIG infusion. The tests were repeated every 3 months, including panel reactive antibody (PRA) testing. Patients were cleared for transplant when they showed a negative or borderline T-cell and B-cell FCXM (less than 20-channel shift difference from the negative control). We evaluated anti-HLA antibody profile before and during IVIG treatment, patient transplant rate, and patient and graft outcomes.

At the time of transplant, all patients received thymoglobulin (6 mg/kg for 4-7 days). Twelve patients (54.5%) also received IVIG at a dose of 2 g/kg on days 0 and 1 postoperatively.

Maintenance immunosuppression consisted of prednisone and tacrolimus for 100% of the cases. Four patients (18.2%) used mycophenolate mofetil and 18 (81.8%) used methyl methanesulfonate as antiproliferative drugs during maintenance.

All patients received cytomegalovirus prophylaxis with intravenous ganciclovir or valganciclovir adjusted for kidney function for 3 months.

Mean patient follow-up was 60.5 (SD, 36.8) months after transplant.

We could evaluate the number of donor-specific antibodies (DSAs), immunodominant DSA (iDSA)-mean fluorescence intensity (MFI), and DSA-MFI sum in 13 patients treated from 2010 onwards, as Luminex assays became available in our laboratory only in 2010.

According to our institutional protocol, all patients undergo graft biopsy in the first 2 weeks after transplant. During follow-up, patients undergo a second biopsy if there is worsening of graft function or the presence of proteinuria.

We graded all rejections according to the Banff 2009 classification, which included C4d staining by immunofluorescence or immunoperoxidase techniques.

### Statistical Analysis

Quantitative data (PRA, number of DSAs, and DSA-MFI sum) are expressed as median (range), and we used the nonparametric Wilcoxon test to compare the groups. We set the level of statistical significance at *p* less than 0.05. We estimated patient and graft survival after transplant using the Kaplan-Meier method.

### Ethical Approval

The study was approved by the local Research Ethics Committee (protocol number 1.629.259/ 2016). Given the retrospective nature of the study, informed consent was waived.

## Results

Of the 45 patients initially enrolled in the study, most were women (n = 38; 84.4%), white (n = 35; 77.8%), and underwent hemodialysis (n = 35; 77.8%). The retransplant rate was 31.1% (n = 14). Mean patient age at first visit was 37.7 (SD, 10.3) years.

After IVIG treatment, the median time to transplant was 12.1 (range, 1-42) months.

The results for PRA, number of DSAs, and DSA-MFI sum before and after desensitization are shown in Table 1.

**Table 1:** Panel reactive antibody (PRA) and donor-specific antibody (DSA)-mean fluorescence intensity (MFI) before and after desensitization

Feature	PRE	POST	p-value
PRA CL I n=22	80.5 (61.25-95.25)	62.5 (48.75-77.75)	0.0425
PRA CL II n=22	83 (42.5-94)	68.5 (18-91.75)	0.2188
DSA number n=13*	1 (1-2)	1 (0.5-2)	0.2500
Immunodominant DSA-MFI	5057.5 (2246-11,691.5)	1389.5 (934.25-2492.5)	<0.0001
DSA-MFI sum n=13**	5522 (3967.5-14,095.5)	1975 (603-5510)	0.0002

* /

** Patients in whom the single PRA test (Luminex) was performed.

**Figure 1:**
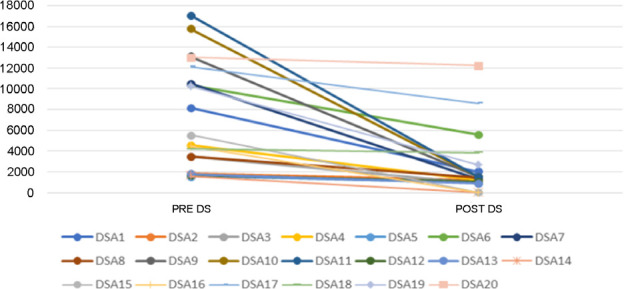
Changes in donor-specific antibody (DSA) after desensitization (DS).

Figure 1 shows the changes in DSAs after desensitization (20 antibodies from 13 patients for whom Luminex was available at the time of analysis). We observed a decrease in MFI in all antibodies analyzed (*p* < 0.0001).

Until being cleared for surgery, patients who underwent a transplant received a median of 6 (range, 3-16) monthly IVIG infusions.

We found no difference in the number of IVIG doses according to the number of DSAs (*p* = 0.2607), class I or class II antibodies (*p* = 0.0514), or DSA-MFI sum (*p* = 0.1241).

Eighteen patients (81.8%) had a positive T-cell and/or B-cell CDCXM, and 4 (18.2%) had only a positive T-cell and/or B-cell FCXM. No difference was found between FCXM+/CDCXM- vs FCXM+/CDCXM+ (6 vs 5; *p* = 0.0667) when analyzing the median class I (65% vs 85%; *p* = 0.5226) and class II PRA (58% vs 83%; *p* = 0.9317), the number of DSAs (1 vs 1.5; n = 0.5686), and DSA-MFI sum before desensitization (4593 vs 6841; n = 0.6121).

After desensitization, we observed a reduction in the median iDSA from 5522 to 1301 (*p* = 0.0002) (Table 2).

**Table 2:** Immunodominant donor-specific antibody (iDSA) before and after desensitization

iDSA	PRE	POST	*p-*value
**A2**	12,089	8617	
**B7**	8160	1993	
**B15**	4472.46	0	
**B 27**	4593	1206	
**B 38**	3463.6	3786.3	
**B 44**	17,053	1500	
**B 51**	1587.8	0	
**DQ 5**	13,000	12,228.6	
**DR 4**	10,499.7	1301.37	
**DR 8**	1792.7	924.6	
**DR 13**	10,337	5579.9	
**DR 16**	5522.9	0	
**DR 17**	3452.89	932.3	
**Median**	5522 (3457.5-11,294)	1301 (462-3786)	0.0002

We considered 4 patients as treatment failures because they could not undergo a transplant during the mean follow-up period of 24.8 (SD, 18.2) months. One patient died after 3 IVIG infusions. One patient remained in desensitization, maintaining a positive CDCXM. Another patient discontinued treatment after 4 years when diagnosed with breast cancer. The last patient discontinued treatment after 13 IVIG infusions due to pregnancy.

Patients who did not undergo a kidney transplant (n = 4) had a median class I PRA of 72.0 (range, 42.7-96.7) and class II PRA of 81.5 (range, 40.5-98.5); no statistically significant difference was observed in transplant recipients. The non-transplanted group had more DSAs (n = 3) than the transplanted group (n = 1), but this difference was not significant (*p* = 0.1646).

The median DSA-MFI sum in patients who did not undergo a transplant was 14,764 (range, 14,661-32,641), which was higher than that in transplant recipients (mean, 5522; range, 3967-14,095), but this difference was not significant (*p* = 0.0926).

**Figure 2:**
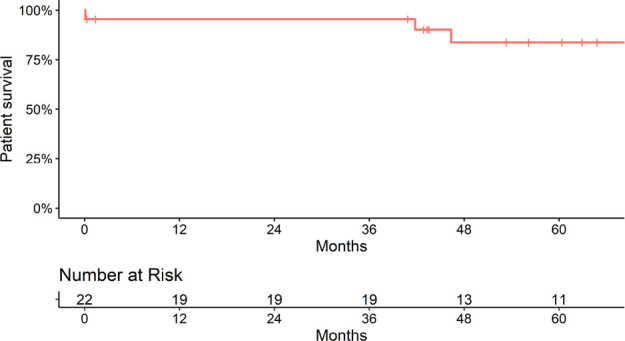
Patient survival.

**Figure 3:**
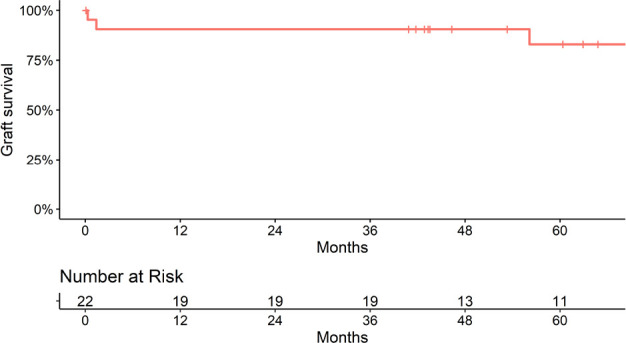
Graft survival.

Patient survival after transplant was 95.5% at 1 year, 95.5% at 3 years, and 83.7% at 5 years (Figure 2). Death-censored graft survival at 1, 3, and 5 years after transplant was 86.4, 86.4, and 79.2%, respectively (Figure 3).

Ten grafts were lost during follow-up, 4 (40.0%) due to chronic antibody-mediated rejection (ABMR). One graft was lost at postoperative day 9 due to arterial thrombosis, and another one was lost at postoperative day 70 after a Banff III T cell-mediated rejection episode. Three patients died with a functioning graft (septic shock, gastroenterocolitis, and respiratory failure), and 1 patient lost the graft after 9 years at another institution due to unknown reasons.

Mean renal function, censored for graft loss and estimated by glomerular filtration rate (calculated by the Modification of Diet in Renal Disease equation), was 66.2 (SD, 14.2) mL/min/1.73 m^2^ at 1 year, 60.4 (SD, 21.2) mL/min/1.73 m^2^ at 3 years, and 60.6 (SD, 22.8) mL/min/1.73 m^2^ at 5 years. Presence of proteinuria was detected in 36.8, 47.4, and 33.3% of patients at 1, 3, and 5 years after transplant, respectively.

Two patients developed tuberculosis, 3 had fungal infections, and 2 had prolonged, chronic diarrhea due to opportunistic germs. Regarding viral infections, 1 patient had cytomegalovirus infection and 2 had polyomavirus infection. One patient had adenovirus-associated hemorrhagic cystitis and 3 had uncomplicated skin varicella-zoster virus reactivation. One patient had genital human papillomavirus infection. Ten patients (50.0%) had no significant infectious complications during follow-up. Of the patients with infectious complications, 6 (60.0%) had no rejection episodes during follow-up.

Neoplasms were uncommon in this group of patients undergoing kidney transplant after desensitization. Only 1 patient had a diagnosis of multiple myeloma after 3.8 years, when she already had advanced chronic graft dysfunction.

## Discussion

Approximately 30% of patients on the waiting list for a kidney transplant in the United States have some degree of sensitization, of whom almost 8000 are highly sensitized, with a PRA above 80%^5^. In Brazil in 2013, 250,621 patients were on the waiting list for a kidney transplant from a deceased donor, of whom 3328 were highly sensitized (PRA > 80%) according to data provided by the National Transplant System. In the same year, 4239 patients underwent a transplant, but only 149 of them were highly sensitized, which corresponds to a transplant rate of only 3.5%^6^.

Given the high levels of anti-HLA antibodies, sensitized patients are less likely to undergo a deceased-donor transplant and more likely to wait longer than non-sensitized patients. According to data from the United Network for Organ Sharing (UNOS) database, the annual transplant rate for sensitized patients is 6.5% against 18% to 20% for non-sensitized patients.

Glotz et al. published in 2002 the results of the first series of patients using IVIG alone for desensitization, achieving a high transplant rate: 86.7% (of a total of 15 treated patients, 13 underwent a transplant)^4^. In our study, we obtained a similar rate of access to transplant: 88.7% (29 patients underwent a transplant after desensitization; 7 of them received a deceased-donor graft).

Orandi et al. published in 2016 a multicenter study with an unprecedented and challenging goal: to show that patients who underwent a living-donor transplant after desensitization had longer survival than those who did not undergo a transplant or remained on the waiting list for a deceased HLA-compatible donor. The patients were divided into 3 groups: transplant recipients after desensitization (n = 1025), patients who remained on the waiting list or received a transplant from a deceased donor (n = 5125), and patients who remained on dialysis and did not undergo a transplant (n = 5125). Desensitized patients survived longer than those who waited for a deceased-donor transplant and those on long-term dialysis at 1 year (95.0 vs 94.0 and 89.6%), 3 years (91.7 vs 83.6 and 72.7%), 5 years (86.0 vs 74.4 and 59.2%), and 8 years (76.5 vs 62.9 and 43.9%) of follow-up (*p* < 0.001)^7^. In our study, using a desensitization protocol with IVIG alone, we obtained similar patient survival rates at 1 year (95.5%), 3 years (95.5%), and 5 years (83.7%).

Kahwaji et al. conducted a retrospective study of 177 living-donor transplants, of which 66 were of highly sensitized patients subjected to a desensitization protocol that included rituximab, IVIG, and plasmapheresis. The remaining patients were at low immunologic risk. At the end of 6 years of follow-up, survival was 87.9% in sensitized patients and 88.3% in low-risk patients, showing good long-term desensitization results. The incidence of rejection was 30% in sensitized patients and 23% in low-risk patients^8^. In our study, we did not observe ABMR in protocol biopsies performed until postoperative day 7. Throughout the 5-year follow-up, the incidence of ABMR was 27.3%. Possible explanations for the low incidence of rejection in our sample include the fact that patients were cleared for transplant only after achieving a negative FCXM and the use of IVIG as induction therapy.

There is no consensus in the literature on whether DSA-related characteristics (number, intensity, or class) would be linked to the risk of rejection^9^. Phelan et al. published in 2009 a retrospective analysis of 64 living-donor transplant recipients, of whom 12 had DSA at the time of transplant and did not receive thymoglobulin induction therapy. The patients with DSA had no ABMR episodes, and the 2 graft losses in this group were caused by recurrent focal segmental glomerulosclerosis at 35 months and death with a functioning graft at 32 months. Regardless of DSA characteristics, no rejection episodes were observed^10^. In this cases series, neither the number nor the intensity of DSA before transplant was related to a higher risk of ABMR.

Niederhaus et al. associated the intensity of iDSA with a higher risk of rejection^11^. In our analysis, desensitization reduced the patients’ mean iDSA, which was greater than 10,000 MFI in 3 patients. Of these, only 1 did not show a significant reduction in intensity (DQ5: 13,000 > 12,228), but no ABMR occurred in this patient after transplant.

Half of our transplant recipients had no infectious complications. The episodes of infection did not seem to be related to the use of additional immunosuppressive treatment, since 60% of the patients with infection did not have previous rejection episodes.

In our desensitization protocol, the use of IVIG alone did not increase the number of infectious complications. Other drugs commonly used in this process (rituximab, bortezomib, and tocilizumab) are more associated with the development of severe infectious conditions. Interestingly, IVIG has an important anti-infective action owing to the high anti-cytomegalovirus and anti-polyomavirus immunoglobulin rates in its preparation^12^.

The use of IVIG as an exclusive drug for desensitization is an uncommon practice and more studies need to be carried out: our study had a small number of participants and a short observation time, which limits our conclusions.

In summary, we can propose that, after desensitization with IVIG alone, living-donor kidney transplant in these highly sensitized, hard-to-match patients is a safe and effective treatment with an acceptable incidence of rejection and infection, resulting in good long-term patient and graft survival.

## Abbreviations

ABMR: antibody-mediated rejection
CDCXM: complement-dependent cytotoxicity crossmatch
DSA: donor-specific antibody
FCXM: flow cytometry crossmatch
HLA: human leucocyte antigen
iDSA: immunodominant donor-specific antibody
IVIG: polyvalent human intravenous immunoglobulin
MFI: mean fluorescence intensity
PRA: panel reactive antibody
UNOS: United Network for Organ Sharing
